# Ionogel‐Based Electrodes for Non‐Flammable High‐Temperature Operating Electrochemical Double‐Layer Capacitors

**DOI:** 10.1002/cssc.202401874

**Published:** 2025-03-06

**Authors:** Agnese Gamberini, Tobias Burton, Alix Ladam, Ahmad Bagheri, Matteo Abruzzese, Hossein Beydaghi, Valentina Mastronardi, Elena Calcagno, Samaneh Vaez, Alberto Morenghi, Teresa Gatti, Anais Falgayrat, Francesco Bonaccorso, Sebastien Fantini, Sebastiano Bellani

**Affiliations:** ^1^ BeDimensional S.p.A. via Lungotorrente Secca 30R 16163 Genova Italy; ^2^ Solvionic 11 Chemin des Silos 31100 Toulouse France; ^3^ Department of Applied Science and Technology Politecnico di Torino Corso Duca degli Abruzzi 24 10129 Torino Italy; ^4^ Graphene Labs Istituto Italiano di Tecnologia Via Morego 30 16163 Genova Italy

**Keywords:** Electrochemical double-layer capacitors (EDLCs), Ionic liquids (ILs), Electrodes, Ionogels, Temperature

## Abstract

The design of interfaces between nanostructured electrodes and advanced electrolytes is critical for realizing advanced electrochemical double‐layer capacitors (EDLCs) that combine high charge‐storage capacity, high‐rate capability, and enhanced safety. Toward this goal, this work presents a novel and sustainable approach for fabricating ionogel‐based electrodes using a renewed slurry casting method, in which the solvent is replaced by the ionic liquid (IL), namely 1‐ethyl‐3‐methylimidazolium bis(fluorosulfonyl)imide (EMIFSI). This method avoids time‐consuming and costly electrolyte‐filling steps by integrating the IL directly into the electrode during slurry preparation, while improving the rate capability of EDLCs based on pure non‐flammable ILs. The resulting ionogel electrodes demonstrate exceptional electrolyte accessibility and enable the production of symmetric EDLCs with high energy density (over 30 Wh kg^−1^ based on electrode material weight) and high‐rate performance. These EDLCs could operate at temperatures up to 180 °C, far exceeding the limitations of traditional EDLCs based on organic electrolytes (*e. g*., 1 M TEABF_4_ in acetonitrile, up to 65 °C). Ionogel‐type EDLCs exhibit remarkable long‐term stability, retaining 88 % specific capacity after 10000 galvanostatic charge/discharge cycles at 10 A g^−1^ and demonstrating superior retention compared to conventional EDLCs (50 %), while also maintaining 92.4 % energy density during 100 h floating tests at 2.7 V. These electrochemical properties highlight their potential for robust performance under demanding conditions. This study highpoints the practical potential of ionogel‐based electrodes to advance IL‐based EDLC technology, paving the way for next‐generation energy storage devices with high‐temperature and high‐voltage operational capabilities.

## Graphical abstract

Ionogel‐based electrodes, fabricated using an ionic liquid (EMIFSI) via a sustainable slurry casting method, eliminate costly filling steps while enabling high‐performance EDLCs. These devices demonstrate 30 Wh kg^−1^ energy density, superior rate capability and high‐temperature operation up to 180 °C, outperforming conventional EDLCs. A step forward for next‐gen energy storage.

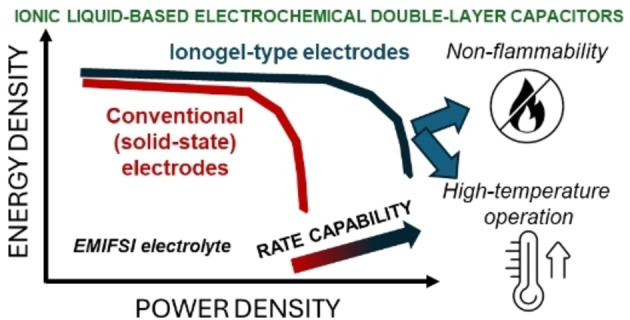

## 1 Introduction

Electrochemical double‐layer capacitors (EDLCs) are attractive electrochemical energy storage (EES) systems that offer high‐power density (>1000 W kg^−1^, >500 W L^−1^)^[1]^ and long cycle life (millions of charge/discharge cycles) complementing battery specifications.^[1]^ Thus, they represent an ideal EES choice in automotive and transportation sectors (*e. g*., driving electronics for internal combustion engine vehicles and electric/hybrid cars,[^2^, ^3^] propulsion systems for electric buses and trams,[^4^, ^5^] regenerative braking systems,[^6^, ^7^] and emergency power units in avionics and trains^[8]^), smart grids (*e. g*., backup generators,[^9^, ^10^] peak shaving,[^11^, ^12^] and load balancing units^[13]^), wind turbines (*e. g*., pitch control systems^[5]^) and power electronics (*e. g*., alternating current filters^[14]^). Nevertheless, their energy density (typically <10 Wh kg^−1^, <8 Wh L^−1^ at cell level for commercial devices) must be increased to conveniently replace battery‐type units, extending their applications in other markets, including flexible and wearable electronics^[15]^ and aerospace sector.[^16^, ^17^] Even though EDLCs are not susceptible to thermal runaway phenomena that arise in other EES systems (*e. g*., metal‐ion batteries^[18]^ and metal‐ion hybrid capacitors^[19]^),[^20^, ^21^] EDLC electrolytes are often formulated using flammable, high‐vapor‐pressure solvents, such as acetonitrile, typically paired with tetraethylammonium tetrafluoroborate (TEABF₄) as the electrolyte salt. Such organic electrolytes can cause internal pressure buildup under temperature variations, restricting the operating temperature window of traditional EDLCs (*e. g*., −40/+65 °C for commercial devices).[^22^, ^23^] In the automotive industry and other applications (*e. g*. wearable electronic devices) wherein stringent safety requirements are imposed (sometimes under harsh environmental conditions) replacing acetonitrile‐based electrolytes is fast becoming a key focus.^[24]^ Towards that goal, ionic liquid (IL) electrolytes have been the centre of intense scientific and industrial attention.^[25]^ In fact, these salts, which are liquid below 100 °C, demonstrate unrivalled safety properties with flash points above 200 °C and negligible vapor pressure, making these compounds essentially non‐flammable. However, their significantly higher viscosities compared to conventional acetonitrile‐based electrolytes limit the performance of IL‐based EDLCs. To overcome this challenge, various approaches have been explored to optimize ion adsorption/desorption processes on electrodes, including ion‐pore size matching and mixing ILs with organic solvents.^[25]^ However, these strategies fail to fully address the existing limitations, emphasizing the need for innovative approaches to overcome these challenges, while preserving the inherent safety and non‐flammability of ILs. To meet the increasingly stringent demands of future energy storage technologies, the development of nanostructured electrode/electrolyte systems with enhanced charge‐storage capabilities, coupled with simplified and scalable manufacturing methods, remains a key focus in the advancement of EDLCs.^[15]^

**Scheme 1:**
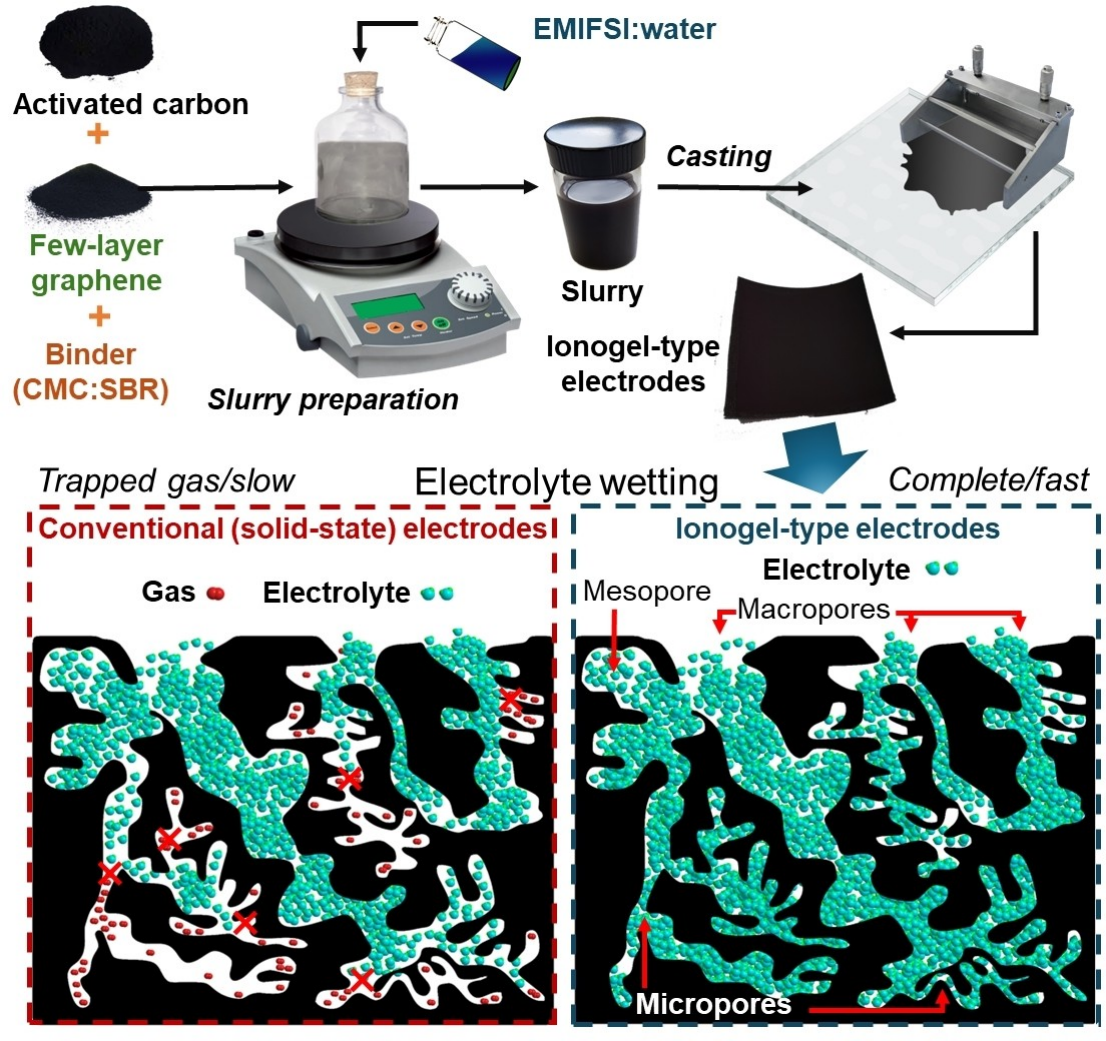
Top: illustration of the production of ionogel‐type electrodes. Bottom: representation of the electrolyte wetting of conventional (solid‐state) and ionogel‐type electrodes.

In this work, we propose a novel approach for producing EDLCs with high energy densities (~30 Wh kg^−1^, referring to the electrode material weight) and with wide operating‐temperature windows (up to 180 °C). As illustrated in Scheme 1, the novelty of this approach relies on the fabrication of electrodes in the form of IL‐based gels, namely ionogels, wherein networks of electrode materials networks enclose large amounts of ILs. The latter are produced on the basis of the slurry casting method, which represents the most common current industrial electrode manufacturing process.^[26]^ In our approach, however, the solvent of the slurry is replaced by IL. Using ILs instead of solvents to produce electrodes and separators for energy storage devices has recently been patented by Solvionic as an effective method for increasing the performance IL‐based devices.[^27^, ^28^] Besides their well‐known characteristics, including chemical, thermal, and electrochemical stabilities, non‐volatility and non‐flammability, our ionogels intrinsically offer exceptional accessibility of the electrolyte to the electrode surface being that the IL was intimately mixed with the electrode materials during the electrode fabrication step. Consequently, the use of ionogels also simplifies conventional EDLC assembly processes by omitting the need for the rigorous electrolyte filling/wetting step. The latter is a time‐consuming and cost‐intensive process of EDLC manufacturing that determines the final device performances.[^29^, ^30^, ^31^, ^32^, ^33^] Conventionally, when assembling EDLCs, the electrolyte must be distributed uniformly into the cell,^[34]^ whilst guarantying the removal of residuals gases.^[29]^ Therein, the aim is to maximize the total electrochemically accessible surface area of the electrodes, thus maximizing the cell energy density.^[35]^ The electrolyte filling step may even be repeated multiple times. However excess electrolyte in the cell must be avoided as that would decrease the gravimetric or volumetric performances of the EDLC. This step can require several days for high‐surface area electrodes,^[36]^ requiring vacuum (and optionally heating) to accelerate electrode soaking in a large‐scale cell manufacturing chains.[^36^, ^37^, ^38^] The electrolyte filling/wetting step may also depend on the cell configuration,[^39^, ^40^] which, for the case of EDLCs, may be prismatic, cylindrical, or pouch formats.^[41]^ In ionogels, IL fills the pore volume of the electrodes, intrinsically eliminating gas entrapment and thus avoiding the need for specific electrolyte filling/wetting steps, which are not yet optimized for advanced electrode materials developed at R&D level. After proving the efficient wetting of the electrode materials with pure IL in our ionogels, symmetric EDLCs were assembled and characterized. These cells demonstrated high energy density combined with high‐rate capability, outperforming devices produced with conventional electrodes that suffer from poor electrolyte wettability. Finally, electrochemical characterization of our EDLCs was evaluated at different temperatures (up to 180 °C) proving that safe power output is possible in conditions in which traditional EDLCs, as well as other EES devices (*e. g*., metal‐ion batteries), fail.^[42]^

## 2 Methods

### 2.1 Materials

Activated carbon powder (AB‐520), styrene‐butadiene rubber (SBR) and carboxymethyl cellulose (CMC) binders, and conductive carbon‐coated aluminium (C‐coated Al) foils were purchased from MTI Corp.

### 2.2 Materials Characterization

Thermogravimetric analysis (TGA) measurements were carried out using a TGA Q500 (TA Instruments) thermogravimetric analyzer in N_2_ flow from 40 °C to 700 °C at a heating rate of 10 °C min^−1^. Gas physisorption measurements were carried out using 3Flex Adsorption Analyzer (Micromeritics). Specific surface area analysis of the active materials was carried out by N_2_ (purity 99.999 %) adsorption at liquid N_2_ temperature (77.3 K). Before measurements, the powder sample was degassed at 250 °C overnight to remove any adsorbed species. The Brunauer, Emmett and Teller (BET) surface area (S_BET_) was calculated from the N_2_ adsorption isotherm using the multi‐point BET method,^[43]^ considering equally spaced points in a relative pressure range (P/P_0_) from 0.01 to 0.30 with a correlation coefficient exceeding 0.999. Micropore analysis was performed by CO_2_ adsorption (purity 99.995 %) at a temperature of 273.5 K. The measurements were performed after N_2_ physisorption measurements, degassing the sample at 200 °C for 1 h. Pore size distribution (PSD) of the sample was calculated by CO_2_ adsorption isotherm using Density Functional Theory (DFT) method^[38]^ in a P/P_0_ range from 0.001 to 0.03.

### 2.3 Electrodes Fabrication

Ionogels were produced by mixing activated carbon, few‐layer graphene (FLG), produced by BeDimensional S.p.A. through wet‐jet milling exfoliation of graphite,[^44^, ^45^, ^46^, ^47^, ^48^] CMC:SBR (1 : 2.75 wt/wt) with a 90 : 5 : 5 weight ratio in a 1‐ethyl‐3‐methylimidazolium bis(fluorosulfonyl)imide (EMIFSI):water (1 : 1 wt/wt) mixture (solid:liquid content ratio ~1 : 3 wt/wt) using a planetary centrifugal mixer, until obtaining a homogenized slurry,[^49^, ^50^, ^51^] in a method adapted from the process developed by Solvionic.^[27]^ The as‐produced slurry was subsequently deposited onto a C‐coated Al foil by doctor blading using a MSK‐AFA−H200 A coater (MTI Corp.). Conventional (solid‐state) electrodes were also produced from aqueous slurry with the same activated carbon:FLG:CMC:SBR weight ratio. It is worth noting that ionogels can also be produced from water‐free slurries using ILs as the liquid media and binders such as polytetrafluoroethylene (PTFE) or polyvinylidene fluoride (PVDF) that, however, are perfluoroalkyl and polyfluoroalkyl substances (PFAS).^[27]^ To eliminate environmentally and/or biologically hazardous chemicals (*e. g*., F‐containing carboxylic acids and/or ammonium lauryl sulphate used as wetting agents to stabilize PTFE aqueous dispersions),[^52^, ^53^] herein non‐PFAS materials were explored, namely CMC:SBR binders and FSI‐based IL (S−F bonds). The electrodes were dried at 70 °C in a vacuum oven (Binder, VD 53‐UL) overnight. The electrodes were then cut into 15 mm‐diameter discs with MSK−T‐07 compact precision disc cutter (MTI Corp.) followed by vacuum drying (100 °C for 3 h with a temperature ramp of 5 °C min^−1^), in a Büchi® B‐585 glass oven connected to V‐300 vacuum pump to remove water residue. The electrodes were then transferred into an Ar‐filled glove box (MBRAUN UNIlab) in which EDLCs cells were then assembled. The mass loading of the electrode materials was ∼4 mg cm^−2^ for better comparison with the specifications of commercially available EDLCs.^[54]^ Contact angle measurements on the prepared IL‐based electrodes demonstrated a contact angle of zero. This result confirms the excellent wettability of the IL electrolyte on the ionogel‐based electrodes.

### 2.4 Assembly of EDLCs

The EDLCs were fabricated by stacking two electrodes, separated by a cellulose separator (Skeleton Technologies) in a Swagelok‐type cell based on 316 L stainless steel pistons, an insulating PTFE‐coated 316 L stainless steel body, and PTFE sealing rings. EMIFSI (99.9 %, Solvionic) was used as the electrolyte. For high temperature (up to 180 °C) tests, EDLCs were also produced in coin cell configuration using 316 L stainless steel CR2032 coin cell case, 316 L stainless steel spring (Belleville washers), and 316 L stainless steel spacers (MTI Corp.). For these cells, grade GF/A glassy fibre separators (Whatman) were used instead of cellulose one (which were used just for a reference coin cell tested at room temperature for comparison purposes). Coin cell sealing was performed with an MSK‐110 hydraulic crimper (MTI corp.) at a pressure of 60 kg cm^−2^. For comparison purposes, reference cells were also assembled with solid‐state electrodes using a standard organic electrolyte in commercial‐like EDLCs, *i. e*., 1 M TEABF_4_ in acetonitrile. This electrolyte was formulated by mixing TEABF_4_ (Sigma Aldrich) with anhydrous acetonitrile (Sigma Aldrich) in the Ar‐filled glove box.

### 2.5 Electrochemical Characterization

Electrochemical measurements of the EDLCs were first performed at room temperature using a potentiostat/galvanostat (VMP3, Biologic) station controlled *via* its own software. Cyclic voltammetry (CV) measurements were performed at various voltage scan rates, from 5 mV s^−1^ to 1000 mV s^−1^, after 10 preconditioning cycles at 100 mV s^−1^. Once the CVs were completed, galvanostatic charge/discharge (GCD) curves were collected at specific currents ranging from 0.2 A g^−1^ to 50 A g^−1^. Hereafter, specific current refers to the I/m_el_ ratio, in which I (A) is the applied current and m_el_ (g) is the mass of a single electrode (excluding its current collector). The specific capacity (C_s_) (A h g^−1^) of the EDLC was used as an universal metric to consider charge storage mechanisms that might deviate from ideal capacitive behaviour,[^49^, ^55^, ^56^] and was calculated from GCD profiles using C_s_=(I×t_d_)/(3600×m), in which I (A) is the applied current, t_d_ (s) is the discharge time, and m (g) is the total mass of the electrodes (excluding current collectors). In addition, the gravimetric capacitance (C_g_) (F g^−1^) of the EDLCs was calculated from GCD profiles using C_g_=(I×t_d_)/(V_r_×m) in which V_r_ is the rated voltage, *i. e*., the max applied voltage (or operational voltage). Noteworthy, C_g_=(C_s_×3600)/V_r_. The discharge energy density (W h kg^−1^), or discharge specific energy (hereafter referred to just as energy density), of the EDLCs was calculated using the integral equation that considers the non‐linearity of galvanostatic discharge profiles.[^49^, ^55^, ^56^] The charge energy density is calculated similarly to the discharge energy density, except that the integral is calculated over the galvanostatic charge profile. The average discharge power density (W kg^−1^), or specific power (hereafter referred to just as power density), of the EDLCs was calculated as average discharge power density=(discharge energy density×3600)/t_d_. The Coulombic efficiency (CE) of the EDLCs was calculated using the ratio between t_d_ to the charge time (t_c_) of the GCD curve, *i. e*., CE=t_d_/t_c_. The energy efficiency (EE) of the EDLCs is given by the ratio between the discharge energy density and charge energy density. Maximum power density (P_max_) of the EDCLs was calculated as P_max_=V_r_^2^/(4×ESR×m), in which ESR is the equivalent series resistance. The latter was estimated from the galvanostatic discharge profile as ESR=▵V_drop_/(2×I), in which V_drop_ is the voltage drop measured at the initial stage of discharge step. The performances of EDLCs in coin cell configuration were also evaluated as a function of the temperature, placing the devices on a temperature‐resistant holder in a mechanical convection oven. Metallic wires were used to bring the electrical contacts of the positive and negative electrodes outside the oven. High‐temperature extension cables (Biologic) were used for precaution. The electrochemical stability of the EDCLs was preliminarily evaluated over 1000 GCD cycles. Afterwards, floating tests (voltage hold protocols) were carried out on the EDCLs to assess their lifetime according to industrial‐like aging protocols, *e. g*., standard IEC62391.[^57^, ^58^] Floating tests were conducted by holding the EDLCs at their V_r_ for 100 h and periodically conducting 5 GCD cycles at 1 A g^−1^ to determine the cell electrochemical performances. More in detail, GCD cycling was performed every 10 h, corresponding to a single floating cycle. Electrochemical impedance spectroscopy (EIS) measurements of the prepared EDLCs were performed in the frequency range from 0.01 Hz to 500 kHz at the discharged state with an AC voltage amplitude of 20 mV.

## 3 Results and Discussion

**Figure 1:**
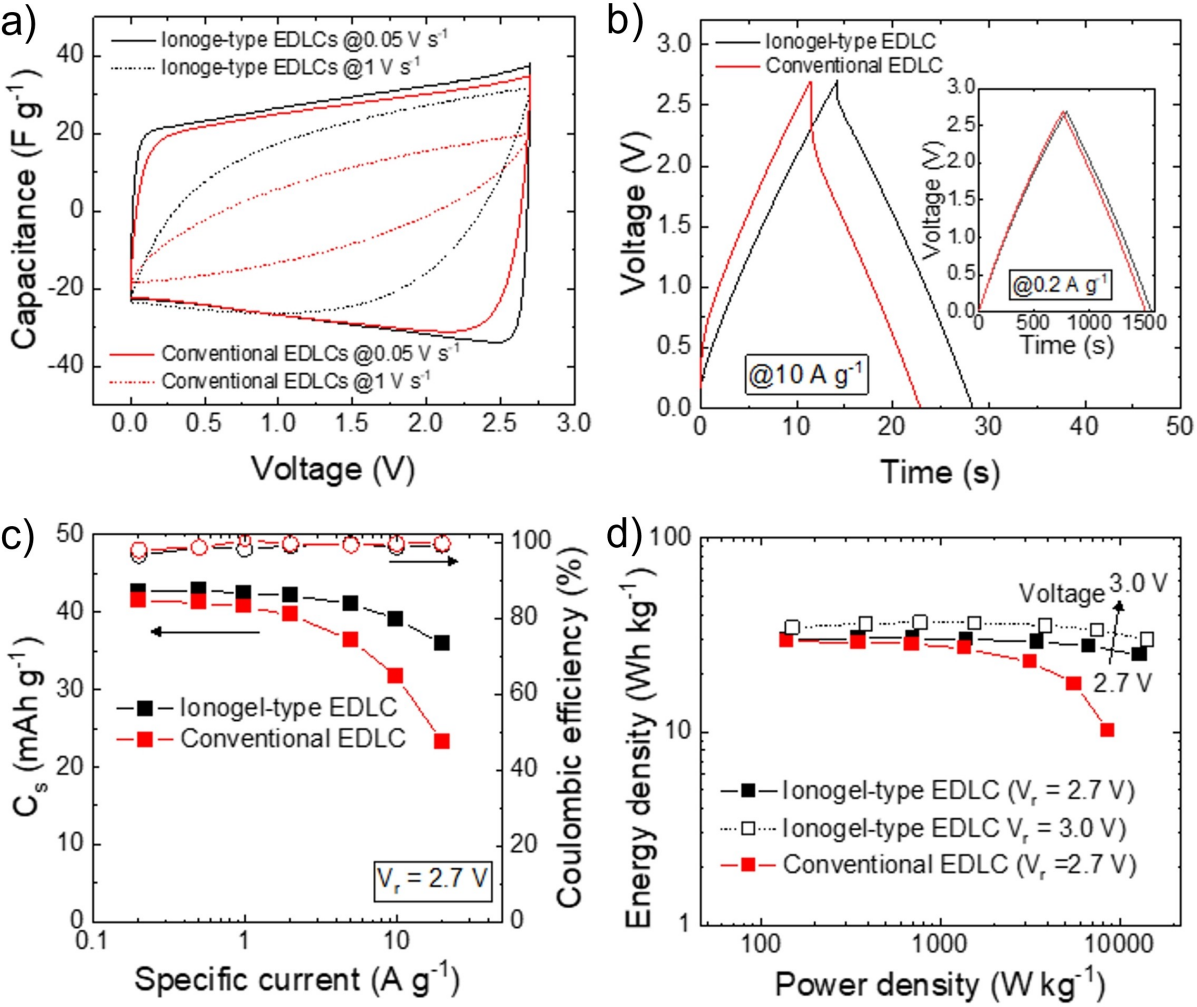
a) CV curves measured for ionogel‐type and conventional EDLCs at voltage scan rates of 0.05 V s^−1^ and 1 V s^−1^. b) GCD profiles measured for ionogel‐type and conventional EDLCs at the specific current of 10 A g^−1^. The inset panel shows the GCD profiles of the investigated EDLCs measured at a specific current of 0.2 A g^−1^). c) C_s_ (left y‐axis) and CE (right y‐axis) *vs*. specific current plots measured for ionogel‐type and conventional EDLCs at V_r_=2.7 V. d) Ragone plots measured for ionogel‐type and conventional EDLCs at V_r_=2.7 V. The Ragone plot measured for ionogel‐type EDLC at V_r_=3.0 V is also shown for comparison. Electrolyte: EMIFSI.

The liquid electrolyte EMIFSI was selected for this study due to its high ionic conductivity, which ranks among the best available ILs. This superior performance is partly attributed to its relatively low viscosity.^[59]^ Furthermore, while the electrochemical stability window of imidazolium‐based electrolytes is narrower than that of pyrrolidinium counterparts, EMIFSI demonstrates stability comparable to state‐of‐the‐art electrolytes.^[60]^ Another reason for selecting this IL is its industrial maturity, as evidenced by Solvionic′s pilot production line with a capacity of 1.5 t/month. Ionogel‐type electrodes were produced by depositing a slurry of electrode materials using EMIFSI as the main liquid media. In the final electrodes, the weight content of the electrode materials (excluding the current collectors) was 60 wt % for EMIFSI, 36 wt % for activated carbon, 2 wt % for FLG and 2 wt % for CMC:SBR. The miscibility of EMIFSI with water has allowed the use cheap water‐soluble binders, namely CMC:SBR. This avoids the use of common F‐containing binders (*e. g*., PTFE and PVDF)^[61]^ that, in addition to being more expensive than aqueous binders, are dispersed in toxic solvents (*e. g*., N‐Methyl‐2‐pyrrolidone ‐NMP‐ for PVDF) or are stably dispersed in water by environmentally and/or biologically hazardous wetting agents (*e. g*., fluorine‐containing carboxylic acids and/or ammonium lauryl sulphate for PTFE).^[52]^ Therefore, our IL‐based ionogels intrinsically eliminate costs associated with the use, drying and recovery of organic solvents (whose content in the slurry can amount to 50–70 wt %),[^27^, ^37^, ^62^] an energy‐intense procedure compared to the low‐temperatures required for (<120 °C, in our case 70 °C with vacuum) drying water‐processed electrodes.[^37^, ^63^, ^64^] The presence of non‐volatile ILs in the slurry ensures that the electrode material surface remains optimally wet by the liquid electrolyte, avoiding gas trapping and the need for time‐consuming electrolyte filling/wetting steps in practical devices.[^27^, ^29^, ^30^, ^31^, ^32^, ^33^] Figure S1 shows the conductivity of EMIFSI as a function of the temperature, from −10 °C to 80 °C. The data confirm that EMIFSI is a high‐conductivity IL, fulfilling essential specifications for high‐power electrochemical energy storage devices.[^65^, ^66^, ^67^] In general, FSI^−^‐based ILs display lower viscosities and higher conductivities than those containing bis(trifluoromethane)sulfonimide (TFSI).[^65^, ^68^, ^69^] These properties can be associated with the small size of FSI^−^ anions (~95 Å^3^, smaller than the size of TFSI^−^ anions, 147 Å^3^)[^70^, ^71^] as well as the flexibility of its structure (–SO_2_−N^(−)^−SO_2_−),[^70^, ^71^] leading to weak van der Waals interactions in the corresponding electrolyte systems.[^70^, ^71^] To prove the validity of our approach, symmetric EDLCs were assembled with ionogel‐type electrodes made of 90 wt % of activated carbon (relative to the solid content) and using pure EMIFSI as the non‐flammable electrolyte. The electrochemical performances of the ionogel‐based EDLCs were measured with a V_r_ of 2.7 V and compared with those of reference devices containing solid‐state electrodes produced using the aqueous slurry casting method (see details in Methods). Figure 1a shows the comparison of the CV curves (plotted as C_g_ *vs*. voltage) measured for the investigated EDLCs at two representative voltage scan rates, *i. e*., 0.05 V s^−1^ and 1 V s^−1^. The ionogel‐type EDLCs shows higher C_g_, especially at the highest voltage scan rate of 1 V s^−1^ in which the conventional EDLC exhibits a leaf‐shaped CV curve. The latter can be associated to resistive losses, including those resulting from the electrolyte transport into porous electrodes. Figure 1b reports the GCD profiles measured for the EDLCs at specific currents of 10 A g^−1^ and 0.2 A g^−1^, confirming the electrochemical behaviour observed from CV data. At the high specific current of 10 A g^−1^, the ionogel‐type EDLC has a lower ▵V_drop_ than the conventional EDLC. In fact, the ESR of the ionogel‐type EDLC is reduced by 30 % when compared to that of the conventional EDLC, thus enabling high‐power operations even in the presence of pure IL as the electrolyte. Consequently, the ionogel‐type EDLC exhibits a P_max_ of 479.6 kW kg^−1^ that is significantly superior to that of conventional EDLC (139.3 kW kg^−1^). The CV curves and GCD profiles measured for these EDLCs at various voltage scan rates and specific currents, respectively, are reported in the Supporting Information (Figure S2–S5). Noteworthy, the CV curves of the EDLCs feature specific currents that increase with increasing voltage, leading to butterfly‐like shapes. The latter are commonly observed for devices based on active carbonaceous materials,[^72^, ^73^] and have been associated with the dependence of the quantum capacitance of graphitic materials as a function of the voltage associated with a potential‐dependent density of state (*i. e*., their charge carrier density increases with the position of the Fermi level within the density of state).[^72^, ^73^] Consistently with CV data, the GCD profiles show quasi‐triangular shapes, with voltage‐dependent slopes of the GCD profiles, indicating voltage‐dependent capacitance. Figure 1c shows the rate capability of the EDLCs wherein C_s_ and the CE are plotted as a function of the specific current. Importantly, C_s_ was plotted as a universal metric instead of C_g_ to consider the non‐linearity of the GCD profiles.^[74]^ Despite the deviation from an ideal capacitive behaviour, the EDLCs exhibit CEs approaching 100 %, excluding Faradaic effects. Figure 1d shows the Ragone plots (energy density *vs*. power density) measured for the investigated EDLCs, as calculated using the GCD data, illustrating the superior rate capability of the ionogel‐type EDLCs compared to that of conventional EDLCs. At the power density of 12.96 kW kg^−1^, the ionogel‐type EDLC retains ~83 % of the energy density measured at the lowest power density of 0.14 kW kg^−1^. Contrarily, the energy density of conventional EDLCs drops by 65.4 % when increasing the power density from 0.14 kW kg^−1^ (29.2 Wh kg^−1^) to 8.67 kW kg^−1^ (10.1 Wh kg^−1^). The Ragone plot obtained for ionogel‐type EDLCs operating at a V_r_ of 3.0 V is also shown, confirming the high‐rate capability of this device configuration together with remarkable energy densities higher than 30 Wh kg^−1^ (*e. g*., 36.6 Wh kg^−1^ at 0.77 kW kg^−1^). By normalizing the energy of the EDCLs by the weight of the active material only, a metric often reported in literature,^[75]^ energy densities as high as 40.7 Wh kg^−1^ are obtained for ionogel‐type EDLCs. The CV and GCD data recorded for ionogel‐type EDLCs operating at V_r_ of 3.0 V are reported in the Supporting Information (Figure S6‐S7). For comparison purposes, the electrochemical characterization of a conventional EDLC using a standard organic electrolyte (1 M TEABF_4_ in acetonitrile) is also reported in Supporting Information (Figure S8). Importantly, despite the use of a more viscous electrolyte, the proposed ionogel‐type EDLC shows performances (C_s_, energy density, CE/EE, and rate capability) comparable to those of conventional EDLCs based on commercial‐like organic electrolyte, which is however flammable and not able to operate at high temperatures (*e. g*., ≥70 °C).

**Figure 2:**
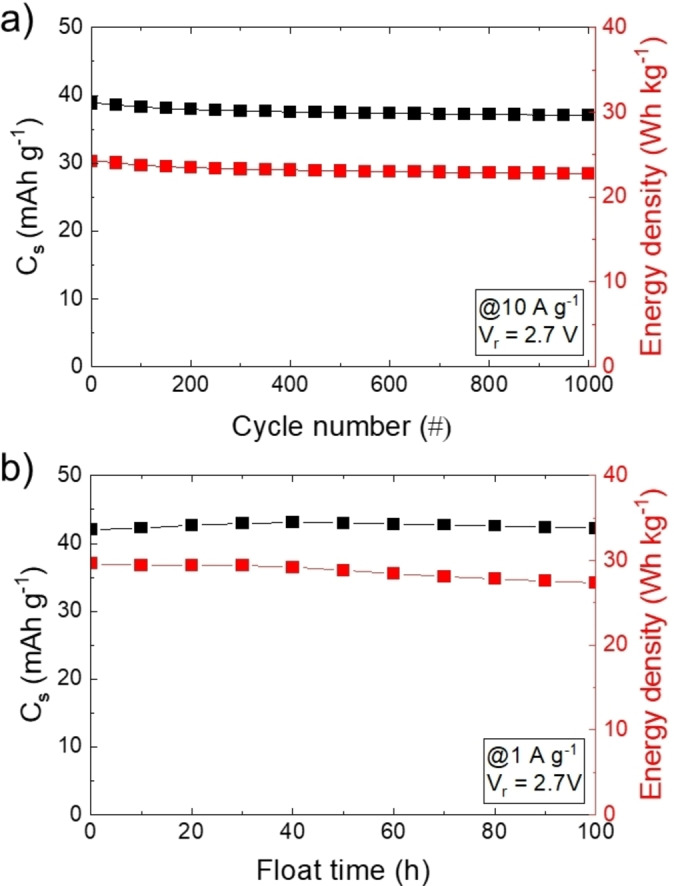
a) Cyclic stability of the ionogel‐type EDLC: C_s_ (left y‐axis) and energy density (right y‐axis) *vs*. cycle number plots measured at 10 A g^−1^ and V_r_=2.7 V. b) Floating stability of the ionogel‐type EDLC: C_s_ (left y‐axis) and energy density (right y‐axis) *vs*. float time plots measured at 1 A g^−1^ and V_r_=2.7 V. Electrolyte: EMIFSI.

Considering the non‐solid‐state form of our ionogel‐type electrodes, it is crucial to evaluate possible drawbacks related to the stability of the electrode/current collector interfaces. The electrochemical stability of the devices was preliminarily evaluated over GCD cycling, checking a relatively limited number of cycles, *i. e*., 1000, before moving towards more demanding industrial‐like protocols, *i. e*., floating tests.^[57]^ Figure 2 shows the GCD cycling of the ionogel‐type EDLCs operating at V_r_ of 2.7 V over 1000 cycles at 10 A g^−1^, showing satisfactory energy density retention (93.8 %) even superior to that of conventional EDLC (84.9 %) (Figure S9a). The cyclic instability of the latter may be associated to the high viscosity of the electrolyte, *i. e*., pure EMIFSI (24.5 cP at 25 °C^[76]^ *vs*. ~0.2 cP for 1 M TEABF_4_ in acetonitrile (ACN)^[77]^), which may encounter difficulties in accessing the surface area of porous active materials of EDLCs operating at high specific currents, causing electromechanical stresses. The investigated EDCLs also demonstrated a satisfactory performance stability during the floating test at V_r_ of 2.7 V, retaining 100 % of the initial C_s_ and 92.4 % of the initial energy density (measured at 1 A g^−1^). Similar behaviour was recorded for the conventional EDLC (Figure S8b).

To exploit the combination of high‐rate capability combined with the use of EMIFSI as a non‐flammable electrolyte, the characterization of our ionogel‐type EDLCs was extended to high temperatures ranging from 25 to 180 °C. In case of temperature‐related damage, coin cell systems were chosen for these tests as they are much more affordable compared to Swagelok systems. Also, glassy fiber separators were used instead of cellulose ones to ensure that all the cell components were thermally and dimensionally stable up to temperatures as high as 200 °C, even though the large thickness of the former (0.26 mm) results in EDLCs with high ESRs (*ca*. an order of magnitude higher than those obtained with cellulose separators). TGA measurements of the cell components are reported in Figure S10.

**Figure 3:**
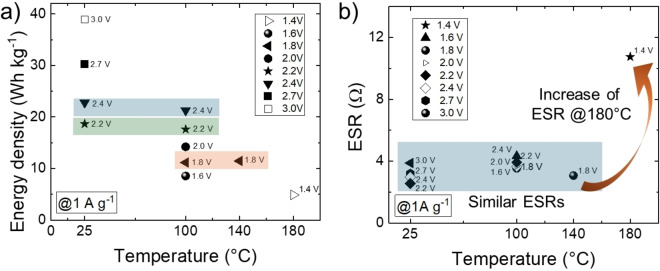
a) Energy densities and b) ESRs of ionogel‐type EDLCs at various temperatures, extrapolated from GCD profiles measured with a specific current of 1 A g^−1^ and increasing progressively V_r_ until devices operated with CE >90 %. Electrolyte: EMIFSI.

Preliminary tests in EDCLs with coin cell configurations using cellulose separators revealed very similar performances (energy density and rate capability) compared to Swagelok‐type devices (Figure S11) whilst also confirming that the ionogel‐type EDLCs significantly outperform conventional EDLCs when using EMIFSI as the electrolyte. Nevertheless, the cyclic stability of EDLCs in coin cell configurations operating at V_r_ of 2.7 V was inferior to that of devices in Swagelok cell configuration (Figure S12 *vs*. Figure 2a), likely due to the lower pressure applied to the electrode/separator/electrode stack, leading to a progressive increase of the ESR, as also observed over prolonged cycling at a conservative V_r_ of 2.4 V (Figure S13). Discrepancies between the lifetimes of different prototypes come down to cell design, as discussed in relevant literature.^[57]^ When using FSI^−^‐based electrolytes, FSI^−^ anions can attack the passivating oxide layer of the positive aluminium current collectors generating insoluble Al‐FSI compounds ‐Al(FSI)_3_‐.[^78^, ^79^, ^80^] This effect is well‐known in Li‐ion batteries, affecting the cathode current collector at potentials higher than 4.0 V *vs*. Li/Li^+^.[^78^, ^79^, ^81^, ^82^] This effect is however less studied in EDLCs even though the upper potentials reached by the positive electrodes are comparable to those at which Li‐ion battery cathodes operate.^[80]^ In this context, the increase of stack pressure can effectively reduce performance fade by keeping an intimate contact between the electrode material and current collector, thus suppressing electrode delamination.[^83^, ^84^] Since the FSI^−^‐induced aluminium corrosion and other parasitic reactions such as electrolyte solvent/salt decomposition and electrode material modifications,^[80]^ are exacerbated when increasing the temperature,^[79]^ our electrochemical tests were conducted by progressively decreasing V_r_ as the temperature increased, with the aim of ensuring sufficient cyclic stability. The electrochemical characterizations of ionogel‐type EDLCs operating at 100 °C and 140 °C were measured with maximum V_r_ values of 2.4 V and 1.8 V, respectively (see Figure S14‐S15), at which no degradation was observed during these GCD analysis. At 180 °C and with a V_r_ value of 1.6 V (Figure S16), the devices underwent degradation during GCD analysis when the specific current was decreased from 20 A g^−1^ to 1 A g^−1^. Based on this preliminary performance assessment, GCD analysis was then performed with a specific current of 1 A g^−1^, increasing progressively V_r_ until device exhibited insufficient CE (<90 %), *i. e*., showing significant parasitic reactions. Figure 3 reports the energy density of the EDLCs measured for different V_r_ and temperature combinations. Ionogel‐type EDLCs exhibit a nearly temperature‐independent energy density for fixed V_r_. Even though the conductivity of the IL increases with increasing temperature, ionogel‐type electrodes intrinsically offer optimal electrolyte accessibility without showing a temperature‐dependent behaviour of the energy density measured at 1 A g^−1^. Importantly, devices exhibited similar ESRs at temperatures below 180 °C (Figure 3b), which again supports an interesting temperature‐independent behavior. However, the ESR recorded at 180 °C increased significantly (*ca*. three times more) compared to those measured at lower temperatures. The increase of the ESR at such elevated temperatures can reasonably be attributed to the deterioration of the current collector/electrode material interface at the positive electrode, as caused by FSI^−^‐induced corrosion, which is exacerbated with the increase of the temperature.^[79]^ Prospectively, strategies that avoid parasitic reactions could be implemented, thus eliminating the need of a reduced V_r_ at high temperatures and yielding very stable ionogel‐type EDLCs. Examples of strategies include the optimization of the electrode mass ratio to limit the maximum potential of the positive electrode^[85]^ and the use of Al‐passivating additives,[^80^, ^86^] as well as the use of other ILs less corrosive than EMIFSI.

**Figure 4:**
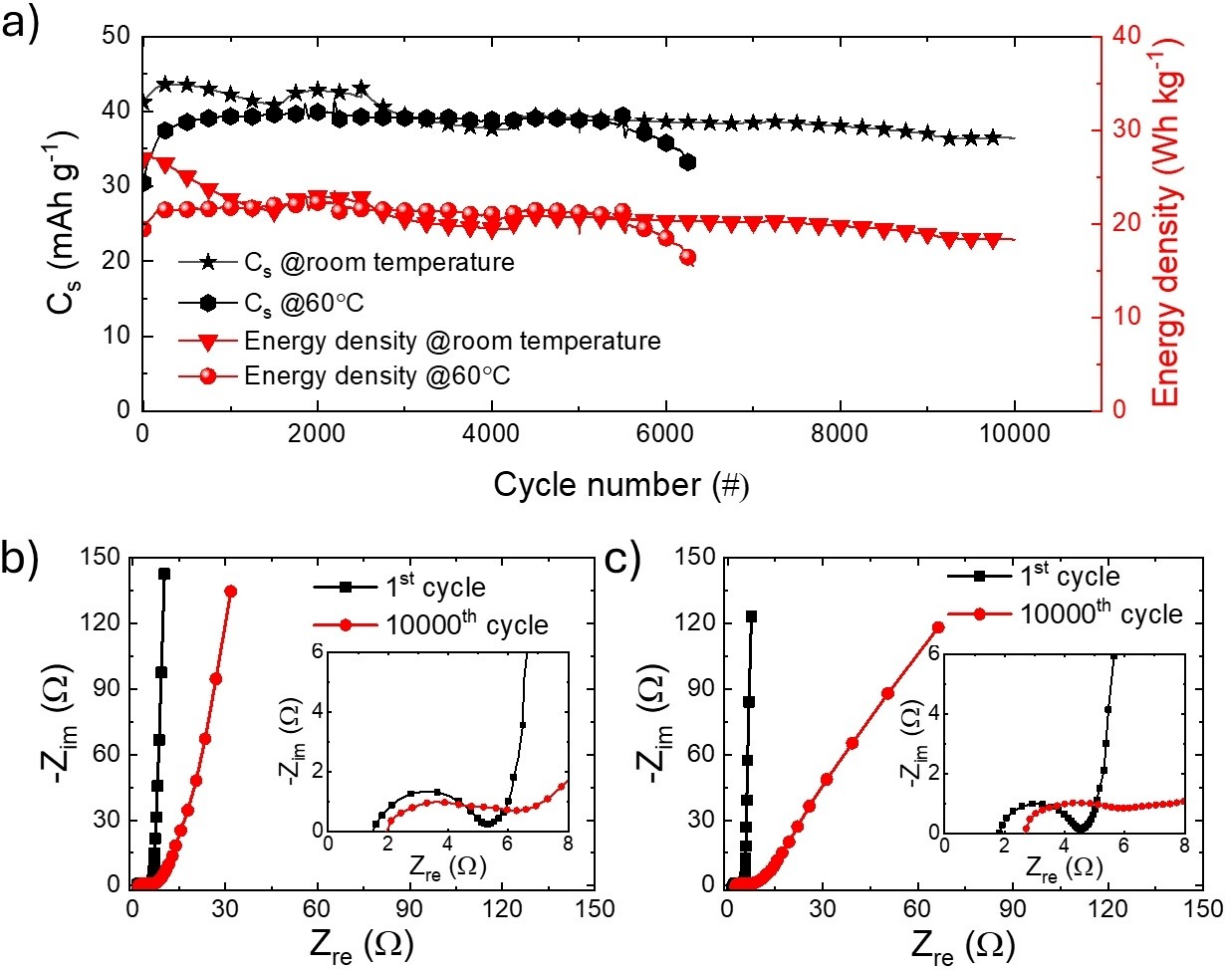
Electrochemical stability of ionogel‐type EDLCs in coin cell configuration operating at room temperature and 60 °C with a V_r_ of 2.7 V. a) Cyclic stability of ionogel‐type EDLCs: C_s_ (left y‐axis) and energy density (right y‐axis) *vs*. cycle number plots measured at 10 A g^−1^. Nyquist plots of the investigated ionogel‐type EDLCs before and after GCD cycles at b) room temperature and c) 60 °C.

To further evaluate the electrochemical performance and thermal stability of ionogel‐type EDLCs operating with V_r_ of 2.7 V, GCD cycles and EIS were performed at room temperature and 60 °C. At room temperature, the electrodes demonstrated excellent cyclic stability, maintaining almost 90 % of their initial C_s_ over 10000 cycles at 10 A g^−1^ (Figure 4a). This stability is attributed to the preservation of the electrode structure, stable ion‐accessible surface area, and minimal thermal stress on the electrolyte, ensuring a stable electrode/electrolyte interface and efficient ion transport. Conversely, at 60 °C, the electrodes displayed reduced stability, possibly linked to FSI^−^ related corrosion, highlighting the need for long‐term anticorrosion strategies to enable practical applications. The EIS analysis provided valuable insights into the electrochemical behaviour before and after GCD cycling. At room temperature (Figure 4b), the Nyquist plot exhibited near‐ideal EDLC characteristics, with a small semicircle in the high‐frequency region indicating low charge transfer resistance (R_ct_). In the low‐frequency region, the ionogel‐type device demonstrated near‐vertical behaviour, signifying excellent capacitive performance. Prolonged cycling resulted in a slight increase in the semicircle diameter, indicating a marginal rise in R_ct_, consistent with the performance retention observed in Figure 4a. After cycling, minor deviations from the vertical line and increased Warburg impedance suggest modest changes in ion diffusion processes within the ionogel‐based electrodes. At 60 °C (Figure 4c), the as‐fabricated EDLCs have shown Nyquist plot with a smaller semicircle in the high‐frequency region compared to the room‐temperature case, indicating minimal R_ct_ and efficient ion transport. However, after 6000 cycles, the semicircle became more pronounced, reflecting a performance degradation accelerated by temperature. The low‐frequency region initially displayed ideal capacitive behavior, but deviations post‐cycling indicated an increase of the ion diffusion resistance, which we ascribed to FSI^−^‐induced corrosion effects, a challenge that we are now working on to address market requirements.

Figure S16a illustrates the stability of an EDLC based on EMIFSI electrolyte but with conventional electrodes. This device retained only ~50 % of its C_s_ after 10000 cycles, indicating a lower cyclic stability compared the ionogel‐type EDLCs. Figure S16b shows the Nyquist plot of this devices, highlighting larger R_ct_ compared to that resulting from ionogel‐type electrodes. The large R_ct_ associated with conventional electrodes is linked to high viscosity of EMIFSI, leading to a poor electrolyte wetting of the electrode surface area. In the low‐frequency region, a Warburg tail reflects hindered ion diffusion. Overall, this analysis further remarks the benefits of using ionogel‐type electrodes in EDLCs based on advanced IL‐based electrolyte with superior safety (*e. g*., non‐flammability). Table S1 compares the performance metrics of the prepared ionogel‐type EDLCs with those reported in relevant literature.

## 4 Conclusions

In summary, we propose a novel approach to manufacture EDLC electrodes relying on the use of ILs as the liquid media for the slurry preparation. These electrodes can be produced using the conventional casting method used for the massive preparation of EDLC electrodes at industrial levels without any change. The resulting electrodes present in the form of ionogels wherein the electrolyte has exceptional accessibility to the electrode surface due to the intimate mixing of the IL with the electrode materials in the slurry state. Prospectively, our ionogel‐type electrodes simplify the fabrication of EDLCs by avoiding the rigorous electrolyte filling/wetting steps, which are time‐consuming and cost‐intensive.[^29^, ^30^, ^31^, ^32^, ^33^] After producing ionogel‐type electrodes with a representative IL (EMIFSI), symmetric EDLCs were assembled using EMIFSI as a non‐flammable and thermally stable electrolyte. Room temperature electrochemical characterization demonstrated that our ionogel‐type EDLCs exhibited high energy density (over 30 Wh kg^−1^) combined with high‐rate capability, outperforming devices produced with conventional electrodes. Lastly, electrochemical characterization of our ionogel‐type EDLCs was performed at temperatures up to 180 °C, demonstrating the feasibility of very high‐temperature operations. This technology opens the door to new types of high energy density applications operating in harsh conditions in which traditional EDLCs based on organic electrolytes (*e. g*., 1 M TEABF_4_ in acetonitrile) fail. Prospectively, our approach can be extended to other types of IL‐based electrolytes. Additionally, further development of the individual components (including the electrolyte, current collectors, active materials, and binder) for high‐temperature conditions may further enhance the performance of our ionogel‐type EDLCs.

## Supporting Information

Supporting Information, including: CV and GCD measurements for ionogel‐type EDCLs operating with different V_r_ (2.7 V and 3.0 V); electrochemical characterization of conventional EDLCs using a standard organic electrolyte (1 M TEABF_4_ in acetonitrile) and operating with a V_r_ of 2.7 V; cyclic and floating stability of conventional EDLCs using EMIFSI as the electrolyte; TGA profiles measured for EDCL components; electrochemical characterization of EDLCs in coin cell format. ESR of ionogel‐type EDCLs (coin cell) over GCD cycling; electrochemical characterization of ionogel‐type EDCLs operating at high temperature (from 100 °C to 180 °C).

## Notes

A. G., A. B., M. A., H. B., V. M., E. C., A. M., and F. B. are employees of BeDimensional S.p.A., a company that is commercializing 2D materials. T. B., A. L., A. F. and S. F. are employees of Solvionic, a company that produces electrolytic grade ionic liquids for batteries and supercapacitors.

## Author Contributions

A. G. and A. B. led the experimental activities, producing and characterizing the electrodes and EDLCs. A. B. M. A, H. B., V. M., S. V., S.B. supported electrochemical characterization and electrochemical data analysis. E. C carried out TGA measurements. T. B, A. L., A. F. and S. F. conceived the ionogel‐pe electrodes. S. B., S. F. and F. B supervised the activities and acquired the financial support. A. G. and S. B. wrote the original draft. The manuscript was written through contributions of all authors. All authors have given approval to the final version of the manuscript.

5

## Supporting information

As a service to our authors and readers, this journal provides supporting information supplied by the authors. Such materials are peer reviewed and may be re‐organized for online delivery, but are not copy‐edited or typeset. Technical support issues arising from supporting information (other than missing files) should be addressed to the authors.

Supporting Information

## Data Availability

The data that support the findings of this study are available from the corresponding author upon reasonable request.
