# Supplementary material for: Ionogel‐Based Electrodes for Non‐Flammable High‐Temperature Operating Electrochemical Double‐Layer Capacitors
Source: ChemSusChem. 2025 Mar 6;18(10):e202401874. doi: 10.1002/cssc.202401874 (PMC12094152; doi:10.1002/cssc.202401874)
Supplement: Supplementary file 1 — Supporting Information [file CSSC-18-e202401874-s001.pdf]

ChemSusChem

Supporting Information

Ionogel-Based Electrodes for Non-Flammable High-Temperature Operating Electrochemical Double-Layer Capacitors

Agnese Gamberini, Tobias Burton, Alix Ladam, Ahmad Bagheri, Matteo Abruzzese, Hossein Beydaghi, Valentina Mastronardi, Elena Calcagno, Samaneh Vaez, Alberto Morenghi, Teresa Gatti, Anais Falgayrat, Francesco Bonaccorso,* Sebastien Fantini,* and Sebastiano Bellani*

Supporting Information

Ionogel-Based Electrodes for Non-Flammable High-Temperature Operating Electrochemical Double-Layer Capacitors

Agnese Gamberini[a‡], Tobias Burton[b‡], Alix Ladam[b], Ahmad Bagheri[a], Matteo Abruzzese[a], Hossein Beydaghi[a], Valentina Mastronardi[a], Elena Calcagno[a], Samaneh Vaez[a,c], Alberto Morenghi[a], Teresa Gatti[c], Anais Falgayrat[b], Francesco Bonaccorso[a,d], Sebastien Fantini[b,*] and Sebastiano Bellani[a,*]*

[a] BeDimensional S.p.A., via Lungotorrente Secca 30R, Genova, 16163 Italy.

[b] Solvionic, 11 Chemin des Silos, Toulouse, 31100 France.

[c] Department of Applied Science and Technology, Politecnico di Torino, Corso Duca degli Abruzzi 24, 10129 Torino, Italy.

[d] Istituto Italiano di Tecnologia, Graphene Labs, Via Morego 30, Genova, 16163 Italy.

[‡] These authors equally contributed.

Corresponding Author: bellanisebastiano@gmail.com; f.bonaccorso@bedimensional.it; sfantini@solvionic.com;

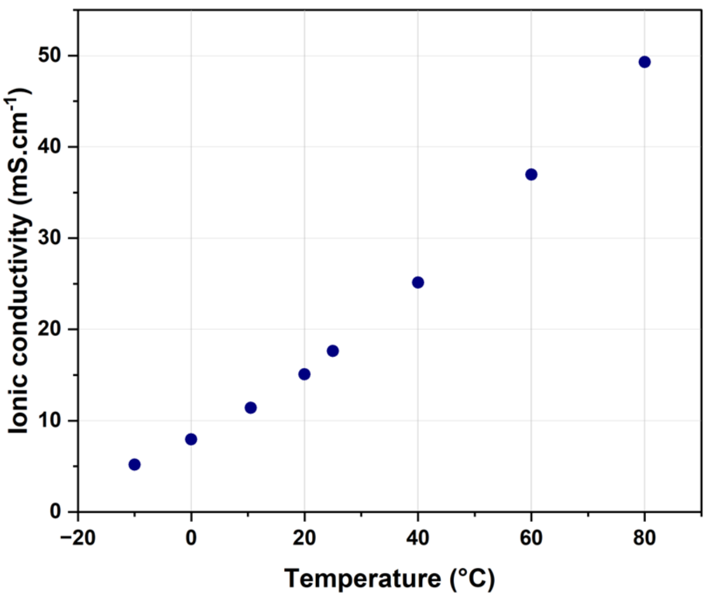

Figure S1. EMIFSI conductivity as a function of the temperature.

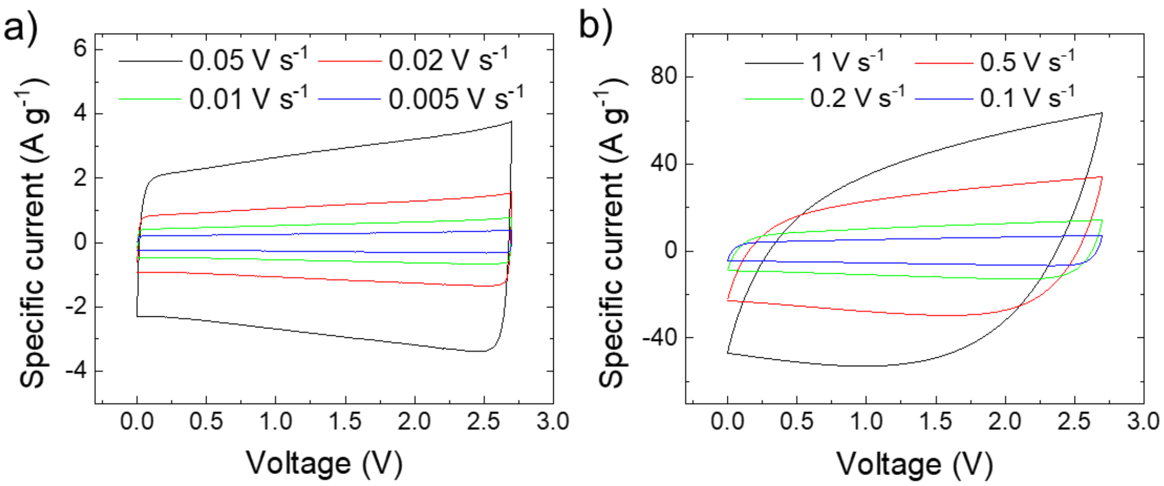

Figure S2. CV curves measured for ionogel-type EDLCs at various voltage scan rates: a) from 0.005 to 0.05 V s^{-1}; b) from 0.1 to 1 V s^{-1} (V_r = 2.7 V; electrolyte: EMIFSI).

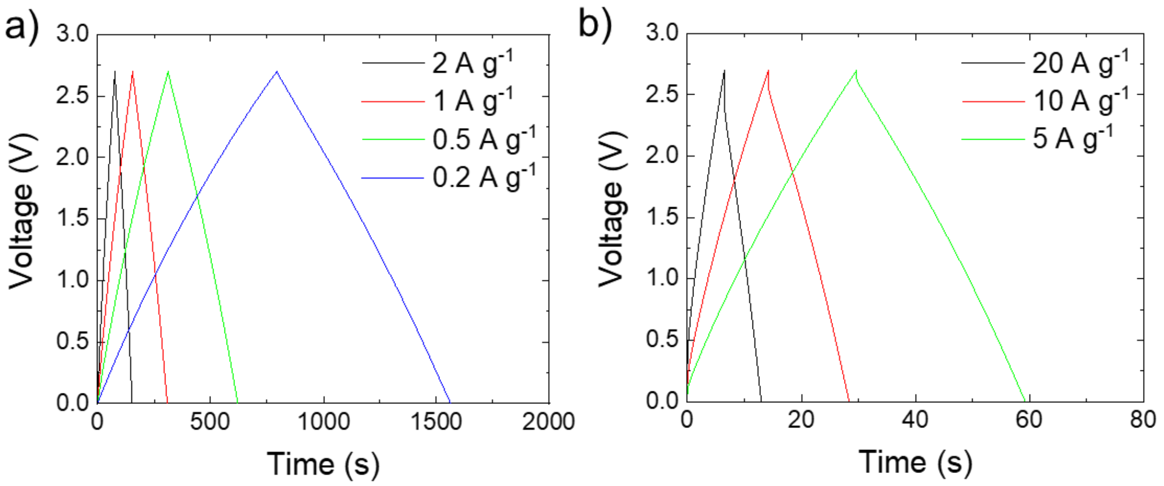

Figure S3. GCD profiles measured for ionogel-type EDLCs at various specific currents: a) from 0.2 to 2 A g^{-1}; b) from 5 to 10 A g^{-1} (V_r = 2.7 V; electrolyte: EMIFSI).

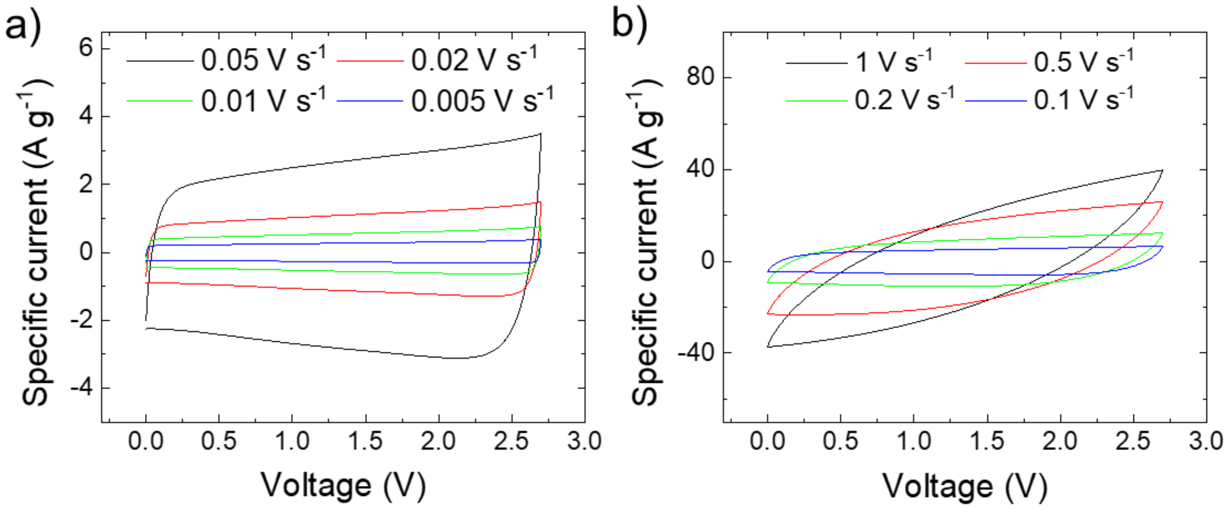

Figure S4. CV curves measured for conventional EDLCs at various voltage scan rates: a) from 0.005 to 0.05 V s^{-1}; b) from 0.1 to 1 V s^{-1} (V_r = 2.7 V; electrolyte: EMIFSI).

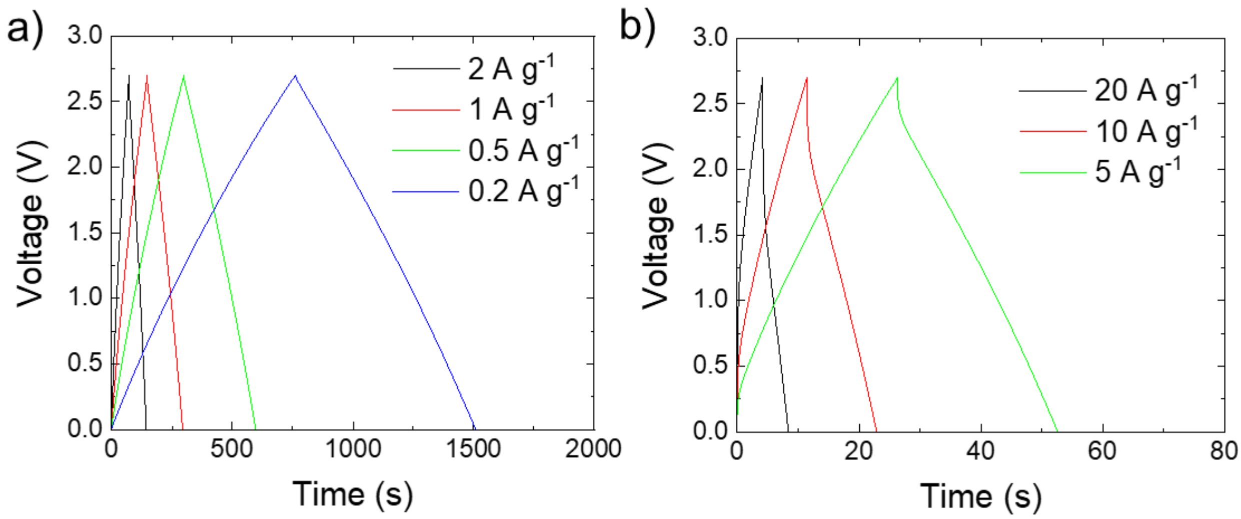

Figure S5. GCD profiles measured for ionogel-type EDLCs at various specific currents: a) from 0.2 to 2 A g^{-1}; b) from 5 to 10 A g^{-1} (V_r = 2.7 V; electrolyte: EMIFSI).

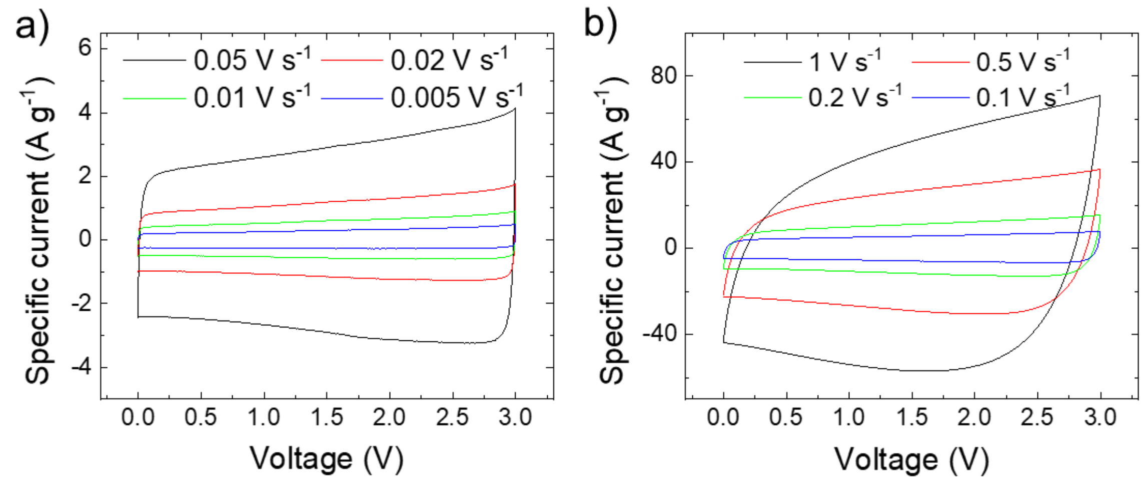

Figure S6. CV curves measured for ionogel-type EDLCs at various voltage scan rates: a) from 0.005 to 0.05 V s^{-1}; b) from 0.1 to 1 V s^{-1} (V_r = 3.0 V; electrolyte: EMIFSI).

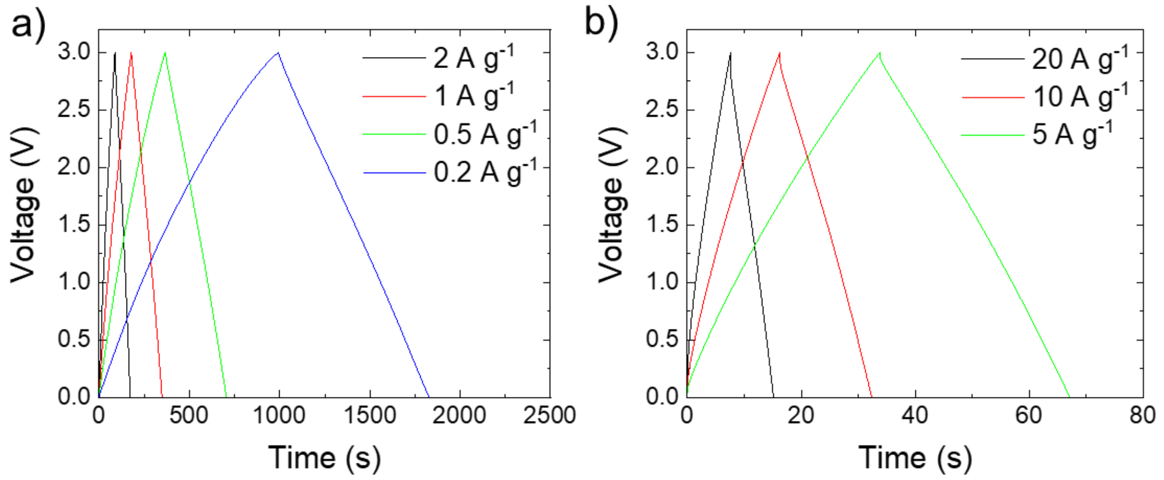

Figure S7. GCD profiles measured for ionogel-type EDLCs at various specific currents: a) from 0.2 to 2 A g^{-1}; b) from 5 to 10 A g^{-1} (V_r = 3.0 V; electrolyte: EMIFSI).

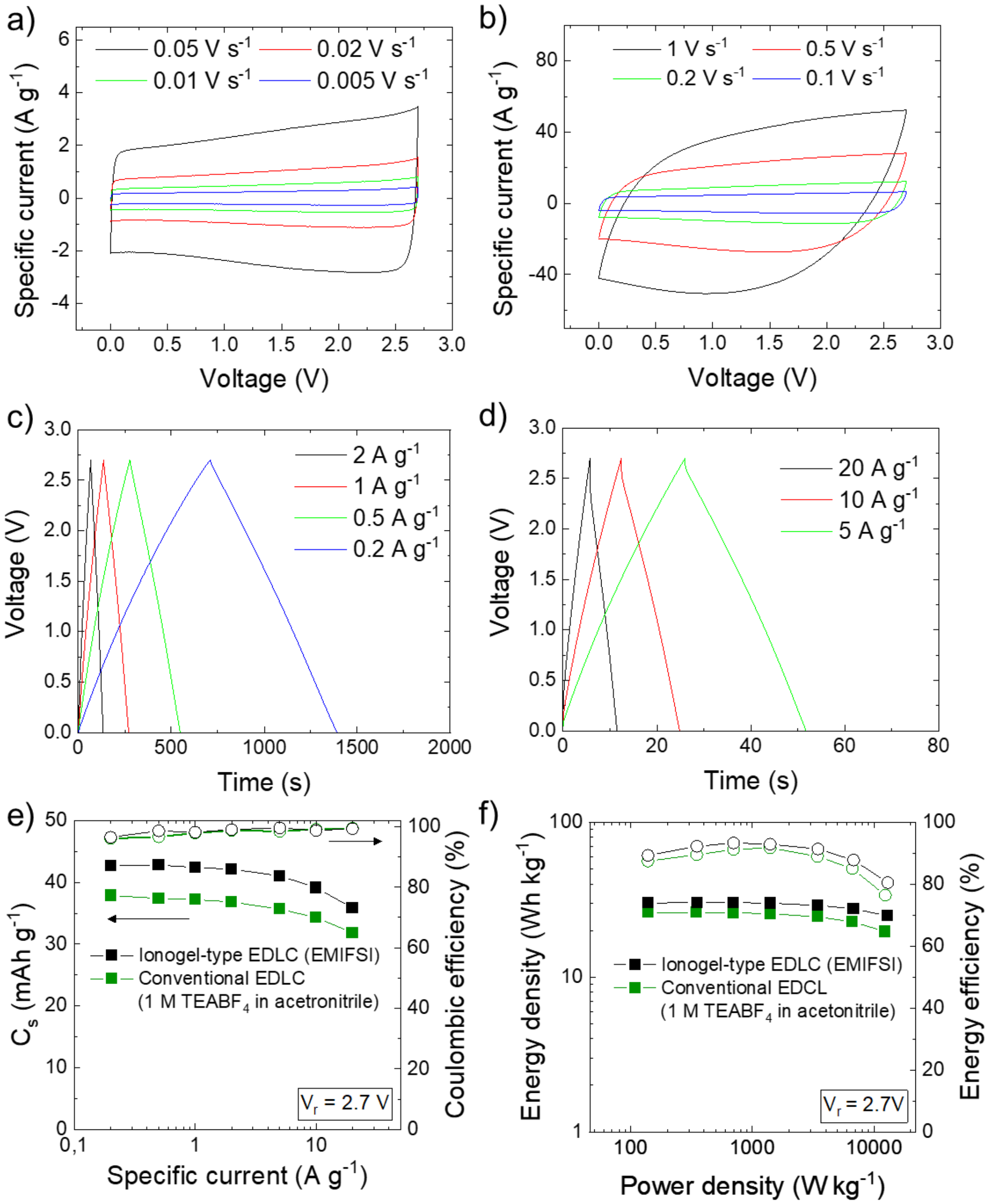

Figure S8. Electrochemical characterization of the conventional EDLC using a standard organic electrolyte (1 M $TEABF_4$ in acetonitrile) and operating with a V_r of 2.7 V. CV curves measured at various voltage scan rates: a) from 0.005 to 0.05 V s^{-1}; b) from 0.1 to 1 V s^{-1}. GCD profiles measured at various specific currents: c) from 0.2 to 2 A g^{-1}; d) from 5 to 10 A g^{-1}. e) C_s (left y-axis) and CE (right y-axis) *vs.* specific current plots measured for the ionogel-type EDLC using EMIFSI as the electrolyte and the conventional EDLC using 1 M $TEABF_4$ in acetonitrile as the electrolyte. d) Ragone plots (left y-axis) and EE *vs.* power density plots (right-axis) measured for the ionogel-type EDLC using EMIFSI as the electrolyte and the conventional EDLC using 1 M $TEABF_4$ in acetonitrile as the electrolyte.

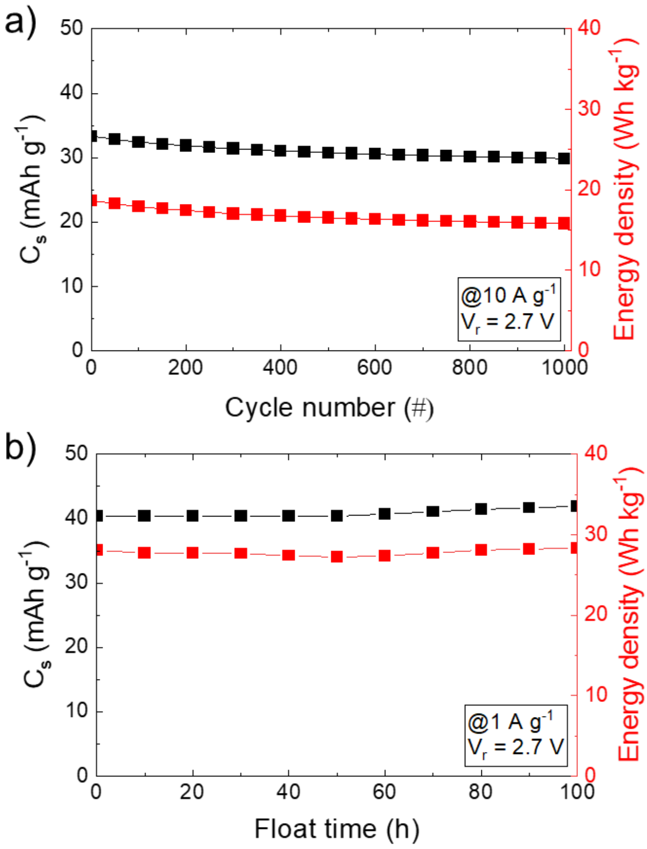

Figure S9. a) Cyclic stability of the conventional EDLC: C_s (left y-axis) and energy density (right y-axis) *vs.* cycle number plots measured at 10 A g^{-1} and V_r = 2.7 V. b) Floating stability of the conventional EDLC: C_s (left y-axis) and energy density (right y-axis) *vs.* float time plots measured at 1 A g^{-1} and V_r = 2.7 V. Electrolyte: EMIFSI.

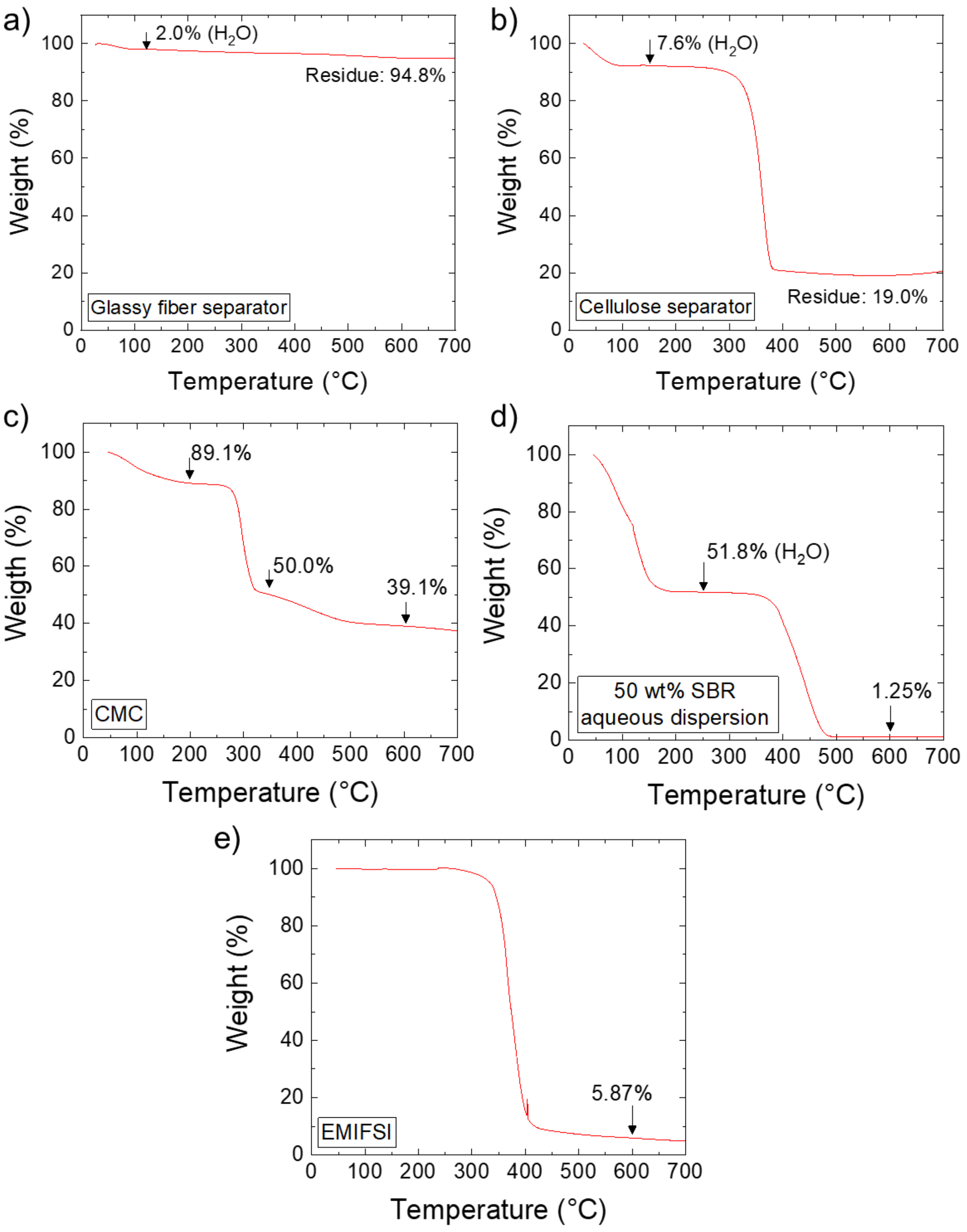

Figure S10. TGA profiles in N_2 measured for: a) glassy fiber separator; b) cellulose separator; c) CMC; d) 50 wt% SBR aqueous dispersion; and e) EMIFSI.

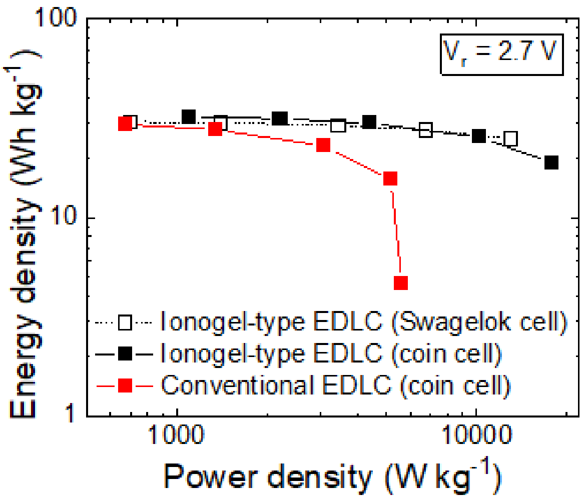

Figure S11. Ragone plots measured for ionogel-type and conventional EDLCs in coin cell formats (V_r = 2.7 V; electrolyte: EMIFSI). The Ragone plot measured for the ionogel-type EDLC in Swagelok cell configuration is also shown for comparison.

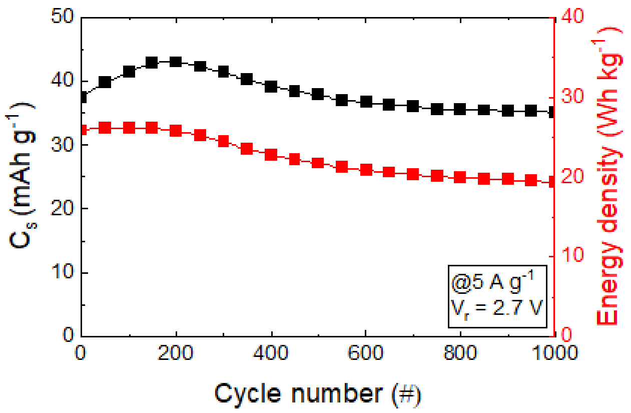

Figure S12. Cyclic stability of the ionogel-type EDLC in coin cell configuration and using a cellulose separator: C_s (left y-axis) and energy density (right y-axis) *vs.* cycle number plots measured at 5 A g^{-1} and V_r = 2.7 V. Electrolyte: EMIFSI. Separator: cellulose.

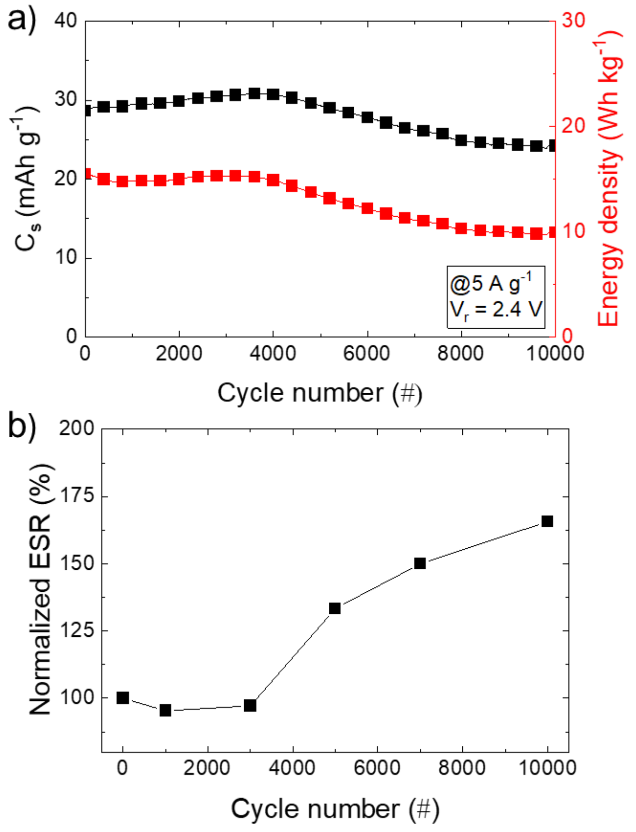

Figure S13. a) Cyclic stability of the ionogel-type EDLC in coin cell configuration: C_s (left y-axis) and energy density (right y-axis) *vs.* cycle number plots measured at 5 A g^{-1} and V_r = 2.4 V. b) Normalized ESR *vs.* cycle number plots. Electrolyte: EMIFSI.

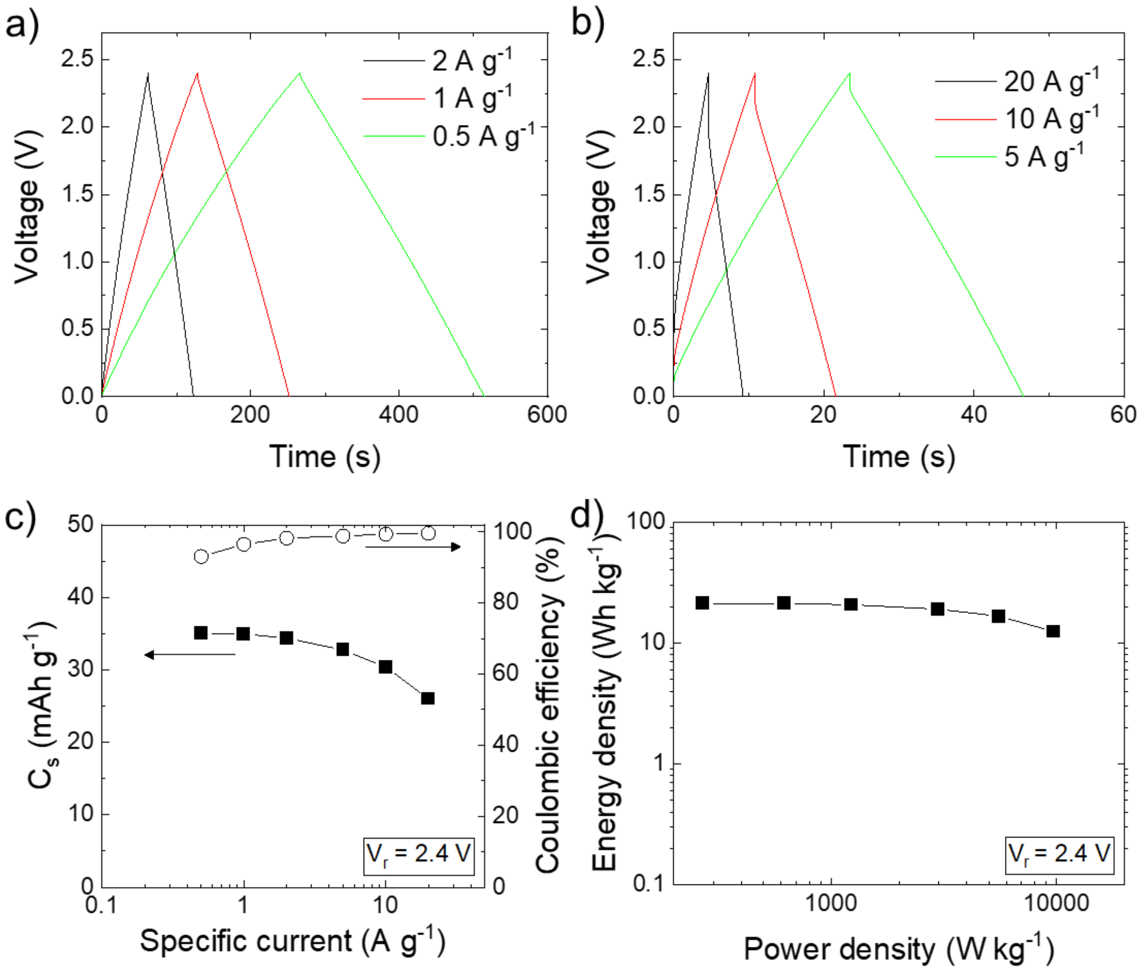

Figure S14. Electrochemical characterization of the ionogel-type EDLC in coin cell configuration operating at 100°C with a V_r of 2.4 V. GCD profiles measured at various specific currents: a) from 0.5 to 2 A g^{-1}; b) from 5 to 20 A g^{-1}. c) C_s (left y-axis) and CE (right y-axis) *vs.* specific current plots. d) Ragone plot.

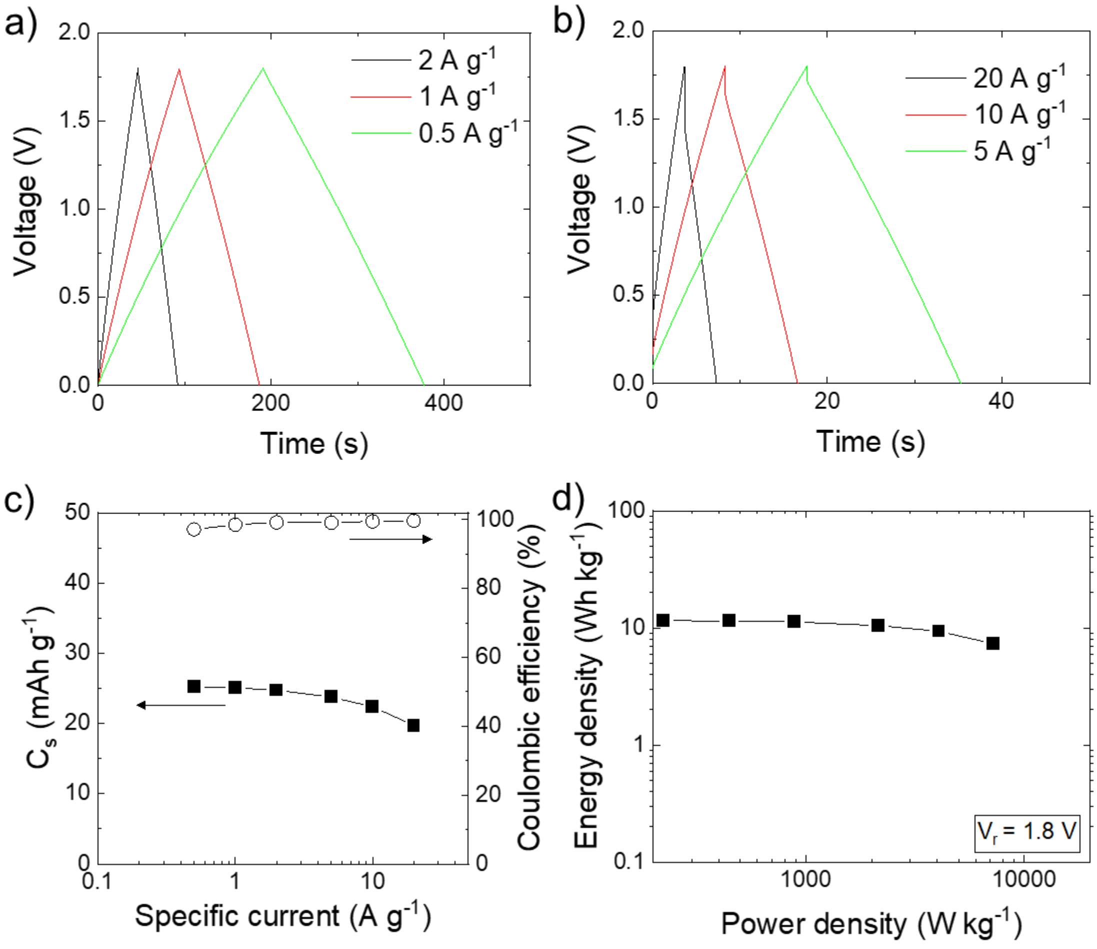

Figure S15. Electrochemical characterization of the ionogel-type EDLC in coin cell configuration operating at 140°C with a V_r of 1.8 V. GCD profiles measured at various specific currents: a) from 0.5 to 2 A g^{-1}; b) from 5 to 20 A g^{-1}. c) C_s (left y-axis) and CE (right y-axis) *vs.* specific current plots. d) Ragone plot.

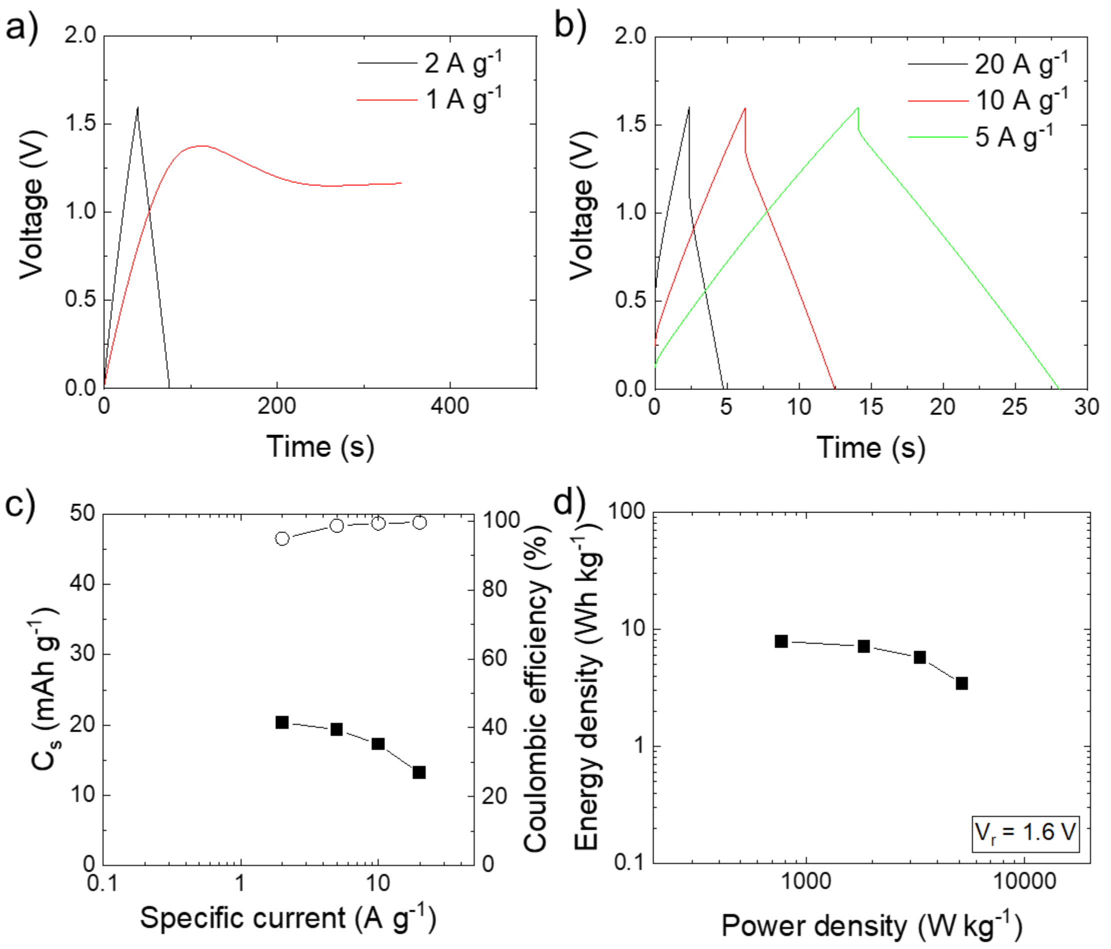

Figure S16. Electrochemical characterization of the ionogel-type EDLC in coin cell configuration operating at 180°C with a V_r of 1.6 V. GCD profiles measured at various specific currents: a) from 1 to 2 A g^{-1}; b) from 5 to 20 A g^{-1}. At 1 A g^{-1}, the presence of parasitic reaction impeded the charging of the EDLC up to 1.6 V. c) C_s (left y-axis) and CE (right y-axis) *vs.* specific current plots. d) Ragone plot.

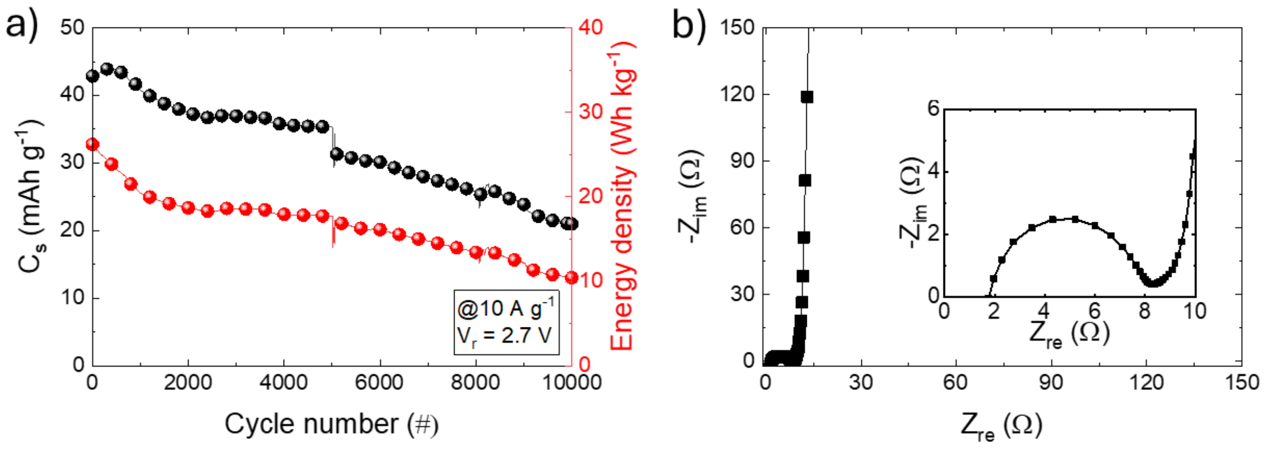

Figure S17. Electrochemical stability of a conventional EDLC in coin cell configuration operating at room temperature with a V_r of 2.7 V. a) Cyclic stability of the conventional EDLC: C_s (left y-axis) and energy density (right y-axis) *vs.* cycle number plots measured at 10 A g^{-1}. b) Nyquist plots of the as-fabricated conventional EDLC. The inset panels show the enlargement of the high-frequency region of the Nyquist plot.

Table S1. Comparison of the EDLC performance reported in this work with that of EDLCs various electrolytes, including ILs, from the literature. AC: activated carbon; FLG: few-layer graphene; CB: carbon black.

Electrodes	Electrolyte	C_g (I_s)	E_s (P_s)	Voltage window	Ref
Ionogel-type AC:FLG	EMIFSI	113.7 F g^{-1} (1 A g^{-1})	36.5 Wh kg^{-1} (1.4 kW kg^{-1})	3 V	This work
Ionogel-type AC:FLG	EMIFSI	113.1 F g^{-1} (1 A g^{-1})	30 Wh kg^{-1} (1.4 kW kg^{-1})	2.7 V	This work
Conventional AC:FLG	EMIFSI	108.6 F g^{-1} (1 A g^{-1})	26.9 Wh kg^{-1} (1.4 kW kg^{-1})	2.7 V	This work
Conventional AC:FLG	1 M $TEABF_4$ (ACN)	99.2 F g^{-1} (1 A g^{-1})	25.7 Wh kg^{-1} (1.4 kW kg^{-1})	2.7 V	This work
Mesoporous carbon	EMITFSI	9 F g^{-1} (0.5 A g^{-1})	19.4 Wh kg^{-1} (220 W kg^{-1})	3 V	[1]
Mesoporous carbon	$EMIBF_4$	14 F g^{-1} (0.5 A g^{-1})	22.9 Wh kg^{-1} (0.24 kW kg^{-1})	3 V	[1]
Asparagus waste-derived AC	7 M KOH (aq)	145 F g^{-1} (1 A g^{-1})	6.5 Wh kg^{-1} (185 W kg^{-1})	1.2 V	[2]
Mangosteen shell-derived AC:CB	EMIFSI	112.8 F g^{-1} (0.5 A g^{-1})	28.2 Wh kg^{-1} (0.51 kW kg^{-1})	2.8 V	[3]
Mangosteen shell-derived AC:CB	2.5 M KNO_3 (aq)	114.1 F g^{-1} (1 A g^{-1})	10.1 Wh kg^{-1} (570 W kg^{-1})	1.6 V	[3]
RGO	$BMIPF_6$	45 F g^{-1} (10 mV s^{-1})	18.9 Wh kg^{-1} (2.8 kW kg^{-1})	3 V	[4]
Reed-derived AC:CB	$EMIMBF_4$	147 F g^{-1} (1 A g^{-1})	45 Wh kg^{-1} (750 W kg^{-1})	3 V	[5]
Microporous AC	EMIFSI	115 F g^{-1} (1 A g^{-1})	42 Wh kg^{-1} (780 W kg^{-1})	3 V	[6]
Microporous AC	PMPFSI	92 F g^{-1} (1 A g^{-1})	45 Wh kg^{-1} (900 W kg^{-1})	3.5 V	[6]
P[DABA][H_2SO_4]-derived AC	$EMIBF_4$	183 F g^{-1} (1 A g^{-1})	100 Wh kg^{-1} (1 kW kg^{-1})	4 V	[7]
MWCNTs:AC	P_{4444}FuA	10 F g^{-1} (1 A g^{-1})	29 Wh kg^{-1} (13.3 kW kg^{-1})	3 V	[8]

References

[1] P. F. R. Ortega, G. A. D. Santos, J. P. C. Trigueiro, G. G. Silva, N. Quintanal, C. Blanco, R. L. Lavall, R. Santamaría, *Journal of Physical Chemistry C* **2020**, *124*, 15818–15830.

[2] N. Ahmad, A. Rinaldi, M. Sidoli, G. Magnani, A. Morenghi, S. Scaravonati, V. Vezzoni, L. Pasetti, L. Fornasini, F. Ridi, C. Milanese, M. Riccò, D. Pontiroli, *Journal of Energy Storage* **2024**, *99*, 113267.

[3] V. N. Kitenge, D. J. Tarimo, K. O. Oyedotun, G. Rutavi, N. Manyala, *Journal of Energy Storage* **2022**, *56*, 105876.

[4] Y. Chen, X. Zhang, D. Zhang, Y. Ma, *Materials Letters* **2012**, *68*, 475–477.

[5] D. Zhou, H. Wang, N. Mao, Y. Chen, Y. Zhou, T. Yin, H. Xie, W. Liu, S. Chen, X. Wang, *Microporous and Mesoporous Materials* **2017**, *241*, 202–209.

[6] Q. D. Nguyen, J. Patra, C. Te Hsieh, J. Li, Q. F. Dong, J. K. Chang, *ChemSusChem* **2019**, *12*, 449–456.

[7] L. Miao, H. Duan, Z. Wang, Y. Lv, W. Xiong, D. Zhu, L. Gan, L. Li, M. Liu, *Chemical Engineering Journal* **2020**, *382*, 122945.

[8] I. A. Khan, F. U. Shah, *ACS Sustainable Chemistry and Engineering* **2020**, *8*, 10212–10221.

[9] X. Wang, H. Zhou, E. Sheridan, J. C. Walmsley, D. Ren, D. Chen, *Energy and Environmental Science* **2016**, *9*, 232–239.

[10] X. Xia, J. Zhan, Y. Zhong, X. Wang, J. Tu, H. J. Fan, *Small* **2017**, *13*, 2–9.
